# SIRT1 increases YAP- and MKK3-dependent p38 phosphorylation in mouse liver and human hepatocellular carcinoma

**DOI:** 10.18632/oncotarget.7022

**Published:** 2016-01-25

**Authors:** Yulan Wang, Ran Cui, Xiao Zhang, Yongxia Qiao, Xiangfan Liu, Yefei Chang, Yongchun Yu, Fenyong Sun, Jiayi Wang

**Affiliations:** ^1^ Department of Clinical Laboratory Medicine, Shanghai Tenth People's Hospital of Tongji University, Shanghai, 200072, China; ^2^ Department of Oncology, Shanghai Tenth People's Hospital of Tongji University, Shanghai, 200072, China; ^3^ School of Public Health, Shanghai Jiaotong University School of Medicine, Shanghai, 200025, China; ^4^ Faculty of Medical Laboratory Science, Shanghai Jiaotong University School of Medicine, Shanghai 200025, China; ^5^ Department of Clinical Laboratory Medicine, Third People's Hospital of Yunnan Province, Kunming, 650011, Yunnan Province, China; ^6^ Shanghai Municipal Hospital of Traditional Chinese Medicine Affiliated to Shanghai TCM University, Shanghai, 200071, China; ^7^ Advanced Institute of Translational Medicine, Tongji University, Shanghai, 200092, China

**Keywords:** cell proliferation, liver cancer, RNA polymerase II, promoter, survival

## Abstract

Both oncoprotein and tumor-suppressor activity have been reported for SIRTUIN1 (SIRT1) and p38 in many types of cancer. The effect of SIRT1 on p38 phosphorylation (p-p38) remains controversial and may be organ- and cell-specific. We found that SIRT1 is essential for maintaining liver size and weight in mice. SIRT1 levels were elevated in human HCC compared to adjacent normal liver tissue, and its expression correlated positively with p-p38 levels. Additionally, SIRT1-activated p38 increased liver cancer malignancy. SIRT1 increased phosphorylation and nuclear accumulation of p38, possibly by increasing MKK3 expression. SIRT1 also induced YAP expression, which in turn increased MKK3 transcription. Positive correlations between SIRT1, YAP, MKK3, and p-p38 levels indicate that blocking their activity may prove helpful in treating HCC.

## INTRODUCTION

SIRT1 may act as either a tumor-suppressor or an onco-protein during cancer development. SIRT1 overexpression is sufficient to suppress colon cancer growth [1], but SIRT1 is positively correlated with malignancy in other types of cancers [2]. In HCC, SIRT1 is aberrantly overexpressed and plays an important role in maintaining tumor growth [3-4]. Blocking SIRT1 activity alone or in combination with other therapies has also been suggested as a novel treatment strategy for HCC [5]. Given the importance of SIRT1 in HCC progression, it is important to understand the molecular mechanisms underlying SIRT1-stimulate tumorigenesis in HCC.

The p38/MAPK signaling pathway controls both cell survival and cell death in the initiation and progression of various cancers [6]. To date, four p38 MAPKs that might have overlapping functions have been identified: MAPK14 (p38α), MAPK11 (p38β), MAPK12 (p38γ), and MAPK13 (p38δ). While p38α is abundantly expressed in most tissues, the others seem to be expressed in a more tissue-specific manner [6]. We focused on p38α (hereafter p38) in the present study. Like SIRT1, activation of p38 can either suppress or stimulate tumorigenesis. Several studies support an anti-tumorigenic role for p38; it can induce premature senescence, stimulate p53-depedent growth arrest, and increase the expression of cell cycle inhibitor genes [7-10]. However, enhanced phosphorylation of p38 also serves as a predictor of poor survival in patients with HCC [11]. Moreover, inactivation of p38 can induce mitochondria-mediated apoptosis in HCC cells [12]. These findings demonstrate that p38 may stimulate liver tumorigenesis. However, regulation of p38 specifically in HCC is poorly understood.

Since SIRT1 and p38 are both important for tumorigenesis in HCC, these two proteins may interact with each other. Although SIRT1 overexpression reduces p-p38 in cardiomyocytes under ischemia–reperfusion injury [13], other studies suggest SIRT1 stimulates p-p38 in breast and lung cancer cells as well as in stem cells [14-15]. Currently, the exact relationship between SIRT1 and p-p38 in liver cells and HCC remains unknown.

In the present study, we examined the importance of SIRT1 and p-p38 in mouse liver, human HCC, and established HCC cell lines. We also studied the relationship between SIRT1 and p-p38 in mouse liver and human HCC and investigated underlying mechanisms. These studies clarify the effects of SIRT1 and p-p38 on liver cell proliferation in liver and identify the mechanisms responsible for these effects.

## RESULTS

### SIRT1 affects size, weight, survival, and cell proliferation in mouse liver

**Figure 1:**
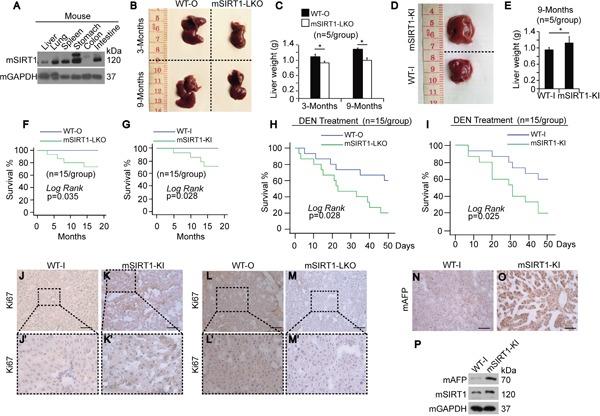
Phenotypes of mice with or without mSIRT1 knock-in or knock-out **A.** Western blots of mSIRT1 in different organs from mice as indicated. **B.** Livers of WT-O and mSIRT1-LKO at 3 or 9 months after birth. **C.** Weight of livers from WT-O and mSIRT1-LKO mice at 3 or 9 months after birth. n=5/group. **D.** Livers of WT-I and mSIRT1-KI mice at 9 months after birth. **E.** Weight of liver from WT-I and mSIRT1-KI mice at 9 months after birth. n=5/group. **F-G.** Survival curve of WT, mSIRT1-LKO, and mSIRT1-KI mice fed with routine diet over a period of 20 months. n=15/group. **H-I.** Survival curve of WT, mSIRT1-LKO, and mSIRT1-KI mice intraperitoneally injected with 50 mg/kg DEN. n=15/group. **J-M.** Representative images of IHC using anti-Ki67 antibodies in liver sections from WT, mSIRT1-KI, and mSIRT1-LKO mice. Boxes in panels j, k, l, and m are enlarged in panels j', k', l', and m', respectively. Scale bar, 500 μm. **N-O.** Representative images of IHC using anti-AFP antibodies in liver sections from WT-I and mSIRT1-KI mice. Scale bar, 500 μm. **P.** Western blots of mAFP in livers from WT-I and mSIRT1-KI mice.

First, we tested mouse SIRT1 (mSIRT1) expression in different organs of C57BL/6J mice. Basal levels of mSIRT1 were lower in liver compared to the other organs (Figure 1A), suggesting that liver may be more sensitive to SIRT1 upregulation. Compared to WT-O mice (Alb-Cre), liver sizes and weights were reduced in liver-conditional mSIRT1 knockout mice (mSIRT1-LKO, Alb-Cre; mSIRT1^*loxp/loxp*^) regardless of time spent feeding (Figure 1B-1C). By contrast, liver sizes and weights were increased in mSIRT1 knock-in mice (mSIRT1-KI, C57BL/6-Actb^*tm3.1(Sirt1)Npa*^/J) compared to WT-I (C57BL/6J) mice (Figure 1D-1E). The above data demonstrate that SIRT1 is essential for maintaining liver size and weight.

Interestingly, both LKO and KI of mSIRT1 in mice led to a shortened life span compared to WT mice over the course of 20 months (Figure 1F-1G), indicating that both decreases and increases in SIRT1 levels shorten longevity. To determine whether SIRT1 affects tumorigenesis, we fed mice low dosages of N-Nitrosodiethylamine (DEN), but all mice, including WT, mSIRT1-LKO, and mSIRT1-KI, died before tumors formed. Notably, both mSIRT1-LKO and mSIRT1-KI mice died sooner than WT mice (Figure 1H-1I), suggesting that SIRT1 dysregulation may result in more rapid death after carcinogen exposure.

Because endogenous SIRT1 levels were positively correlated with liver size and weight (Figure 1B-1E), we hypothesized that SIRT1 may influence liver cell proliferation. To address this, we performed IHC using an antibody against Ki67, a cell proliferation marker. Ki67 staining was more intense in the livers of mSIRT1-KI mice (Figure 1K), and less intense in the livers of mSIRT1-LKO mice (Figure 1M), compared to WT mice (Figure 1J and 1L), suggesting a possible role for SIRT1 in stimulating cell proliferation. AFP is a classic tumor marker is used to diagnose HCC in early stages [16]. IHC showed that mouse AFP (mAFP) was elevated in the livers of mSIRT1-KI mice compared to WT-I mice (Figure 1N-1O). To exclude effects of non-specific background staining in IHC, Western blotting (WB) for mAFP was also performed, and the results confirmed those obtained using IHC (Figure 1P). SIRT1 KO did not significantly reduce liver mAFP expression compared to WT-O livers (data not shown). These results demonstrate that SIRT1 overexpression may promote HCC development.

### Elevated SIRT1 levels in human HCC correlate with p-p38 levels and increase cell proliferation

**Figure 2:**
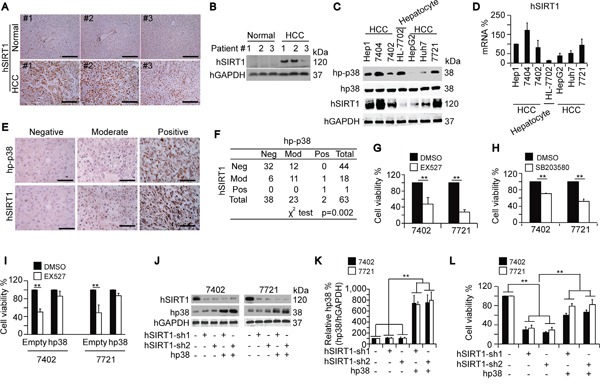
Positive correlation between SIRT1 and p-p38 in human HCC **A-B.** Representative IHC and WB images of hSIRT1 in paired normal liver and HCC tissues. Scale bar, 500 μm. **C.** Western blots of indicated proteins in an established hepatocyte line and HCC cell lines. **D.** The hSIRT1 mRNA expression in different cell lines as measured by qPCR. The GAPDH mRNA was used for normalization of hSIRT1 levels in the qPCR. The normalized mRNA levels of Hep1 cells were arbitrarily set to 100 %. **E-F.** TMA of hp-p38 and hSIRT1 in HCC samples. Representative images of IHC are shown in panel E. Data were analyzed using a χ^2^ test and results are listed in panel F. Scale bar, 200 μm. **G-H.** Blocking SIRT1 and p38 by treatment with EX527 (final concentration of 50 μM) and SB203580 (final concentration of 20 μM), respectively, for 5 days inhibited cell proliferation, as shown by an MTT-based analysis in Bel-7402 and SMMC-7721 cells. Initial number of cells: 5,000 cells/sample. **I.** Bel-7402 and SMMC-7721 cells with or without hp38 overexpression were treated with identical amounts of either DMSO or EX527 at a final concentration of 50 μM for 5 days before MTT-based analysis. Initial number of cells: 5,000 cells/sample. **J-K.** Western blots showing the protein levels of hSIRT1 and hp38 in Bel-7402 and SMMC-7721 cells that were either depleted of hSIRT1 by two different shRNAs, or overexpressed hp38. hGAPDH levels were used to verify equal loading. **L.** hSIRT1 depletion-induced reductions in cell proliferation could be reversed by overexpression of hp38 as measured by an MTT-based analysis in Bel-7402 and SMMC-7721 cells. Initial number of cells: 5,000 cells/sample. Cells were cultured for 5 days before final analysis. The data are shown as mean±SD from three independent experiments (including WB but not TMA). **, *p*<0.01 using the Student's *t*-test.

IHC and WB revealed that human SIRT1 (hSIRT1) expression was elevated in HCC compared to paired normal liver tissues (Figure 2A-2B). Furthermore, hSIRT1 expression was higher in all of the established human HCC lines tested than in the transformed hepatocyte line HL-7702 (Figure 2C). These results suggest that SIRT1 is important in human liver tumorigenesis. We then investigated the relationship between hSIRT1 and human p-p38 (hp-p38) levels. hSIRT1 and hp-p38 levels were positively correlated with each other in almost all of the established HCC lines tested (Figure 2C). Because WB is much less sensitive than qPCR, we also tested SIRT1 mRNA levels in established HCC and hepatocyte lines. SIRT1 mRNA levels were positively correlated with SIRT1 protein levels (Figure 2D). Tissue microarray analysis (TMA) of human HCC specimens also showed that hSIRT1 levels were positively correlated with hp-p38 levels (Figure 2E-2F).

Two HCC cell lines, Bel-7402 and SMM-7721, which have been described in our previous studies [17-18], were utilized to test cell proliferation activities by using EX527 and SB203580, a well-established SIRT1 and p-p38 inhibitor, respectively. Application of EX527, a well-established SIRT1 inhibitor [19], reduced cell proliferation compared to the DMSO-treated control (Figure 2G). Similarly, treatment with SB203580, a p-p38 inhibitor, reduced cell proliferation compared to DMSO-treated controls (Figure 2H). To further analyze the impact of SIRT1 and p38 on cell proliferation, we applied EX527 to cells overexpressing human p38 (hp38) and to control cells with normal hp38 expression. The EX527-induced reduction of cell proliferation was much smaller in cells overexpressing hp38 compared to empty vector-treated controls (Figure 2I). Knockdown of hSIRT1 using a specific shRNA in Bel-7402 and SMMC-7721 cells did not alter p38 protein expression (Figure 2J-2K), but depletion of hSIRT1 dramatically suppressed cell proliferation compared to the control (Figure 2L). Interestingly, this inhibitory effect was partially reversed by simultaneous overexpression of p38 (Figure 2J-2L). These results suggest that the pro-carcinogenic functions of SIRT1 in HCC cells might rely on p38 phosphorylation, but not on endogenous p38 expression.

### SIRT1 stimulates phosphorylation of p38

**Figure 3:**
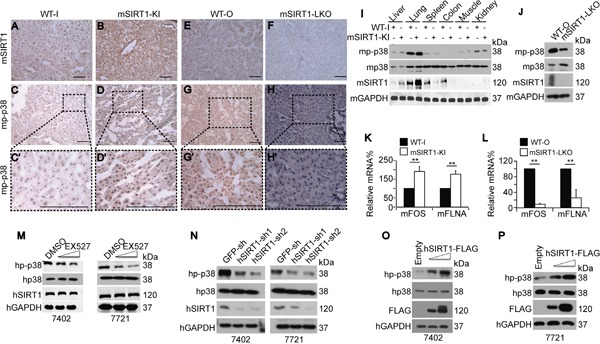
SIRT1 upregulated p-p38 in mouse livers and HCC cells **A-H.** Representative images of IHC staining with anti-SIRT1 and anti-p-p38 antibodies in the livers of WT, mSIRT1-KI, and mSIRT1-LKO mice. Boxes in panels C, D, G, and H are shown enlarged in panels C', D', G', and H', respectively. Scale bar, 500 μm. **I.** Western blots of mp-p38, mp38, and mSIRT1 in different organs in WT-I and mSIRT1-KI mice. **J.** Western blots of mp-p38, mp38, and mSIRT1 in the livers of WT-O and mSIRT1-LKO mice. **K-L.** mFOS and mFLNA mRNA levels in the livers of WT, mSIRT1-KI (K), and mSIRT1-LKO (L) mice, as measured by qPCR. **M.** Western blots of hp-p38, hp38, and hSIRT1 in Bel-7402 and SMMC-7721 cells treated with identical amounts of either DMSO or EX527 at final concentrations from 10 to 50 μM for 24 h before they were harvested for examination. **N.** Western blots of hp-p38, hp38, and hSIRT1 in Bel-7402 and SMMC-7721 cells infected with GFP-sh, hSIRT1-sh1, and hSIRT1-sh2, respectively. **O-P.** Western blots of hp-p38, hp38, and hSIRT1-FLAG (FLAG) in Bel-7402 (O) and SMMC-7721 (P) cells transfected with empty, or increasing concentrations of hSIRT1-FLAG-expressing, plasmids. The data are shown as mean±SD from three independent experiments (including WB). **, *p*<0.01 using the Student's *t*-test.

To confirm the relationship between SIRT1 and p-p38 in mice, we tested mouse p-p38 (mp-p38) levels in WT, mSIRT1-KI, and mSIRT1-LKO mice. Compared to WT mice, mSIRT1 expression (Figure 3A-3B) and nuclear accumulation of mp-p38 (Figure 3C-3D) were increased in mSIRT1-KI mice, whereas mSIRT1 expression (Figure 3E-3F) and nuclear accumulation of mp-p38 (Figure 3G-3H) were decreased in mSIRT1-LKO mice. Interestingly, compared to the WT mice, mSIRT1 KI was successful in the livers, lungs, and kidneys, but not in the spleens, colons, and muscles, of mSIRT1-KI mice (Figure 3I). Moreover, mp-p38 induction following mSIRT1 KI without significant alteration of mp38 was detected only in the liver and not in other organs (Figure 3I), suggesting that the positive correlation between SIRT1 and p-p38 levels may be liver-specific. On the contrary, mp-p38 expression was down-regulated in the livers of mSIRT1-LKO mice compared to WT-O mice (Figure 3J). To further investigate the relationship between mSIRT1 and mp-p38, qPCR was performed to evaluate the expression of two known p38 target genes, FOS and FLNA [20-21]. mSIRT1 KI induced (Figure 3K), while mSIRT1 KO reduced (Figure 3L), mouse FOS (mFOS) and FLNA (mFLNA) levels in the liver compared to WT controls.

We then tested whether hSIRT1 positively regulates hp-p38 in human HCC cells. Increasing concentrations of the hSIRT1 inhibitor EX527 dose-dependently decreased hp-p38 levels compared to DMSO-treated controls, while total hp38 remained unchanged (Figure 3M). Similarly, compared to the control, inhibition of endogenous hSIRT1 expression using two independent specific shRNAs only reduced hp-p38 levels (Figure 3N). By contrast, transfection of increasing concentrations of hSIRT1-FLAG-expressing plasmids dose-dependently increased hp-p38 levels in both Bel-7402 and SMMC-7721 cells compared to empty vector-transfected controls (Figure 3O-3P). Taken together, the *in vitro* and *in vivo* data demonstrate that SIRT1 is capable of inducing p-p38 levels in a liver-specific manner.

### SIRT1 stimulates nuclear accumulation of p-p38

**Figure 4:**
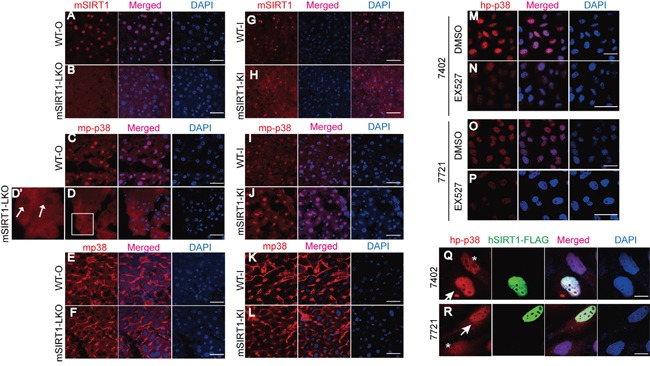
SIRT1 stimulated nuclear accumulation of p-p38 **A-F.** Subcellular localization of mSIRT1 (A-B), mp-p38 (C-D), and mp38 (E-F) in the livers of WT-O and SIRT1-LKO mice, as measured by confocal microscopic experiments. The box in panel D is shown enlarged in panel D'. Scale bar, 100 μm. Arrows indicate nuclear cavities of mp-p38. **G-L.** Subcellular localization of mSIRT1 (G-H), mp-p38 (I-J), and mp38 (K-L) in the livers of WT-I and mSIRT1-KI mice, as measured by confocal microscopic experiments. Scale bar, 100 μm. **M-P.** Subcellular localization of hp-p38 in Bel-7402 (M-N) and SMMC-7721 (O-P) cells treated with identical amounts of either DMSO or EX527 at a final concentration of 50 μM for 24 h. Scale bar, 50 μm. **Q-R.** Subcellular localization of hp-p38 in Bel-7402 (Q) and SMMC-7721 (R) cells with or without successful transfection of hSIRT1-FLAG. Arrows indicate cells successfully transfected with hSIRT1-FLAG, while asterisks indicate cells without successful transfection with hSIRT1-FLAG. Scale bar, 20 μm.

Nuclear accumulation of p-p38 is indicative of its activation [22]; we therefore analyzed the subcellular localization of mp-p38 following mSIRT1 knock-in and -out. As expected, mSIRT1 was absent in the liver of mSIRT1-LKO mice (Figure 4A-4B), and overexpressed in the liver of mSIRT1-KI mice (Figure 4G-4H). Interestingly, compared to WT mice, mSIRT1 KO resulted in the reduction of nuclear mp-p38 (Figure 3C-3D). By contrast, mSIRT1 KI increased nuclear accumulation of mp-p38 (Figure 3I-3J). However, no significant change in subcellular localization of total mp38 was detected in the livers of either mSIRT1-LKO or mSIRT1-KI mice compared to WT mice (Figure 4E-4F and 4K-4L).

In human Bel-7402 and SMMC-7721 cells, nuclear enrichment of hp-p38 was also reduced upon inhibition of SIRT1 by the compound EX527 compared to the DMSO-treated controls (Figure 4M-4N and 4O-4P). By contrast, nuclear accumulation of hp-p38 increased in Bel-7402 and SMMC-7721 cells overexpressing hSIRT1 compared to those without overexpression (Figure 4Q-4R). These data demonstrate that SIRT1 enhances p-p38 expression and stimulates its accumulation in the nucleus.

### SIRT1 increases expression and phosphorylation of MKK3

**Figure 5:**
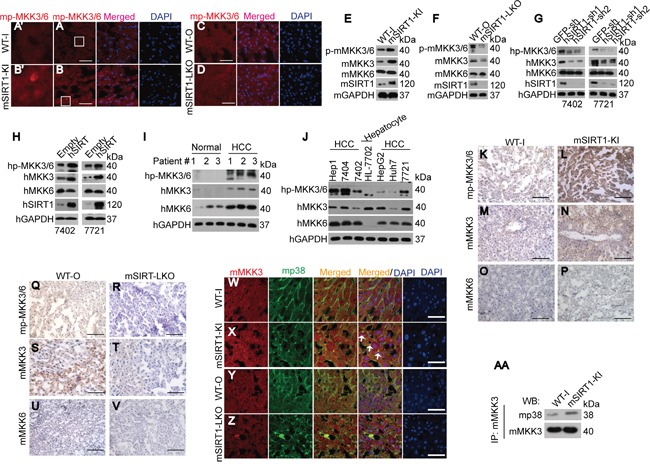
SIRT1 increased p-MKK3/6 by increasing MKK3 levels **A-D.** Subcellular localization of mp-MKK3/6 in the livers of paired WT-I and mSIRT1-KI mice (A-B) and paired WT-O and mSIRT1-LKO mice (C-D). The boxes in panels A and B are shown enlarged in panels A' and B'. Scale bar, 200 μm. **E-F.** Western blots of mp-MKK3/6, mMKK3, and mMKK6 in the livers of paired WT-I and mSIRT1-KI mice (E) and paired WT-O and mSIRT1-LKO (F) mice. **G-H.** Western blots of hp-MKK3/6, hMKK3, and hMKK6 in control and Bel-7402 or SMMC-7721 cells with hSIRT1 knockdown (G) or overexpression (H). **I.** Western blots of hp-MKK3/6, hMKK3, and hMKK6 in paired adjacent normal liver and HCC samples. **J.** Western blots of hp-MKK3/6, hMKK3, and hMKK6 in established hepatocyte line HL-7702 and HCC cell lines as indicated. **K-V.** Representative images of IHC stained with anti-p-MKK3/6, anti-MKK3, and anti-MKK6 in the livers of paired WT-I and mSIRT1-KI mice (K-P) and paired WT-O and mSIRT1-LKO mice (Q-V). Scale bar, 500 μm. **W-Z.** Co-localization of mMKK3 and mp38 in the livers of paired WT-I and mSIRT1-KI mice (W-X) and paired WT-O and mSIRT1-LKO mice (Y-Z). Scale bar, 200 μm. Arrows indicate mMKK3 and mp38 co-localization signals. **AA.** mSIRT1 increased the interaction between mMKK3 and mp38 as measured by co-IP using anti-MKK3 antibodies followed by WB using anti-p38 antibodies in livers from paired WT-I and mSIRT1-KI mice.

Phosphorylation of p38 is a downstream consequence of MKK3/6 phosphorylation (p-MKK3/6) [23-25]; we therefore hypothesized that SIRT1-stimulated p38 phosphorylation may occur through the stimulation of p-MKK3/6. To address this, we first examined the subcellular localization of mouse p-MKK3/6 (mp-MKK3/6) in livers of WT, mSIRT1-KI, and mSIRT1-LKO mice. mSIRT1 KI stimulated nuclear accumulation of mp-MKK3/6 in the liver compared to WT mice (Figure 5A-5B). However, no changes in mp-MKK3/6 subcellular localization were detected between the livers of WT-O and mSIRT1-LKO mice (Figure 5C-5D). We then tested mp-MKK3/6, mouse MKK3 (mMKK3), and mouse MKK6 (mMKK6) expression in mice. Compared to WT mice, both mp-MKK3/6 and mMKK3 were upregulated in mSIRT1-KI mouse livers (Figure 5E) and were downregulated in mSIRT1-LKO mouse livers (Figure 5F). However, there were no changes in mMKK6 expression among the groups (Figure 5E-5F). Notably, mp-MKK3/6 levels increased proportionally to mMKK3 levels (Figure 5E-5F). Similarly, levels of both human p-MKK3/6 (hp-MKK3/6) and MKK3 (hMKK3) were decreased by hSIRT1 knockdown (Figure 5G) and increased by hSIRT1 overexpression (Figure 5H) in human Bel-7402 and SMMC-7721 cells compared to controls. hMKK6 expression was not changed by either knockdown or overexpression of hSIRT1 (Figure 5G-5H). Furthermore, hp-MKK3/6 and hMKK3 levels were increased more than hMKK6 levels in HCC tissues compared to paired normal liver tissues (Figure 5I). In established HCC cell lines, hp-MKK3/6 levels were positively correlated with hMKK3, but not with hMKK6, levels (Figure 5J). IHC confirmed the positive correlation between mp-MKK3/6 and mMKK3, but not mMKK6, levels in the livers of paired WT-I and mSIRT1-KI mice (Figure 5K-5P) and paired WT-O and mSIRT1-LKO mice (Figure 5Q-5V). These results suggest that SIRT1-induced upregulation of MKK3 may occur prior to the upregulation of p-MKK3/6.

Phosphate transfer from MKK3 to p38 requires that they interact with each other [26]; we therefore examined the co-localization of mp38 and mMKK3 in the livers of WT, mSIRT1-KI, and mSIRT1-LKO mice using confocal microscopic experiments. Compared to WT-I mice, co-localization of mMKK3 and mp38 was increased in the livers of mSIRT1-KI mice (Figure 5W-5X). However, changes were too small to detect differences between the livers from WT-O and mSIRT1-LKO mice (Figure 5Y-5Z). co-IP experiments also indicated that binding of mMKK3 and mp38 was increased in the livers of mSIRT1-KI mice compared to WT-I mice (Figure 5AA). Collectively, these results suggest that SIRT1 not only increases MKK3 phosphorylation, but also increases the binding of MKK3 to p38.

### YAP is required for SIRT1 stimulated-MKK3 expression

**Figure 6:**
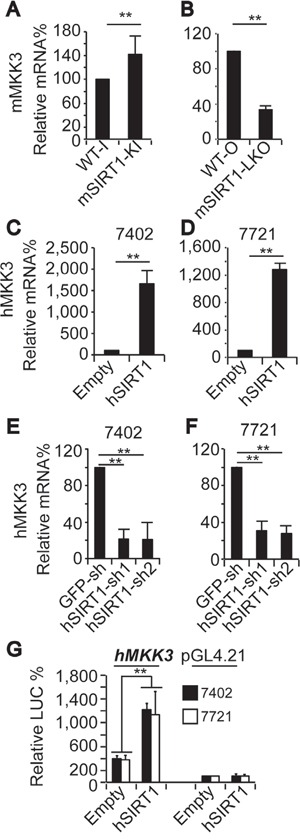
SIRT1 upregulated MKK3 by enhancing its transcription **A-B.** mMKK3 mRNA levels in the livers of paired WT-I and mSIRT1-KI mice (A) and paired WT-O and mSIRT1-LKO mice (B) as measured by qPCR. **C-D.** hMKK3 mRNA levels in Bel-7402 (C) and SMMC-7721 (D) cells infected with empty or hSIRT1 expressing lentivirus, as measured by qPCR. **E-F.** hMKK3 mRNA levels in Bel-7402 (E) and SMMC-7721 (F) cells infected with GFP-sh, hSIRT1-sh1, or hSIRT1-sh2, as measured by qPCR. **G.** Promoter activity from pGL4.21 reporters with or without the promoter region (−1750 ∼+244 nt relative to the transcription start site) of the human *MKK3* gene in Bel-7402 and SMMC-7721 cells with or without hSIRT1 overexpression. The data are shown as mean±SD from three independent experiments. **, *p*<0.01 using the Student's *t*-test.

We then investigated how SIRT1 controls MKK3 expression. A cyclohexemide (CHX) experiment indicated that SIRT1 does not regulate MKK3 protein stability in Bel-7402 and SMMC-7721 cells (data not shown). However, compared to WT, mMKK3 mRNA levels were upregulated by mSIRT1 KI (Figure 6A) and downregulated by mSIRT1 KO (Figure 6B) in mouse liver. In human HCC cells, hMKK3 mRNA levels were also upregulated by hSIRT1 overexpression (Figure 6C-6D) and downregulated by hSIRT1 knockdown (Figure 6E-6F) compared to controls. Luciferase reporter analysis also demonstrated that hSIRT1 increased *hMKK3* promoter activity (Figure 6G).

**Figure 7:**
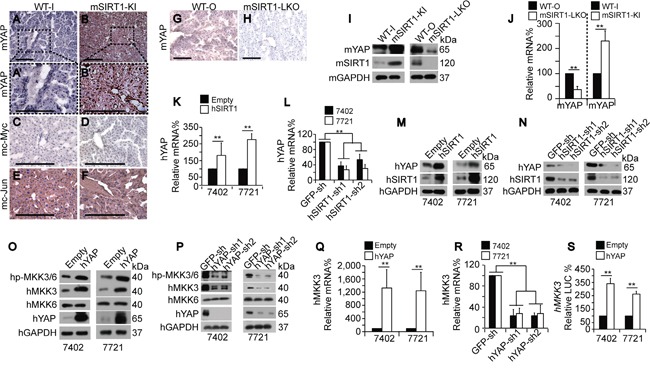
SIRT1 stimulated MKK3 expression through YAP **A-F.** Representative images of IHC for mYAP (A-B), mc-Myc (C-D), and mc-Jun (E-F) in the livers of paired WT-I and mSIRT1-KI mice. The boxes in panels A and B are shown enlarged in panels A' and B'. Scale bar, 500 μm. **G-H.** Representative IHC images of mYAP in the livers of paired WT-O and mSIRT1-LKO mice. Scale bar, 500 μm. **I.** Representative WB images of mYAP and mSIRT1 in the livers of WT, mSIRT1-KI, and mSIRT1-LKO mice. **J.** Relative mYAP mRNA levels in the livers of paired WT-O and mSIRT1-LKO and paired WT-I and mSIRT1-KI mice, as measured by qPCR. **K-L.** hYAP mRNA levels in control and Bel-7402 or SMMC-7721 cells with hSIRT1 overexpression (K) or knockdown (L), as measured by qPCR. **M-N.** Western blots of hYAP and hSIRT1 in control and Bel-7402 or SMMC-7721 cells with hSIRT1 overexpression (M) or knockdown (N). **O-P.** Western blots of hp-MKK3/6, hMKK3, and hMKK6 in control and Bel-7402 or SMMC-7721 cells with hYAP overexpression (O) or knockdown (P). **Q-R.** hMKK3 mRNA levels in control and Bel-7402 or SMMC-7721 cells with hYAP overexpression (Q) or knockdown (R). **S.** Promoter activity from pGL4.21 reporters containing the promoter region (−1750 ∼+244 nt relative to the transcription start site) of the human *MKK3* gene in Bel-7402 and SMMC-7721 cells with or without hYAP overexpression. The data are shown as mean±SD from three independent experiments (including WB). **, *p*<0.01 using the Student's *t*-test.

As promoter activity is largely controlled by transcription factors [27], we then tried to identify a transcription factor that might mediate SIRT1-induced upregulation of MKK3. The proto-oncoprotein mouse YAP (mYAP) might play such a role and was upregulated in the livers of mSIRT1-KI mice compared to WT-I mice (Figure 7A-7B). Additionally, the expression of two other proto-oncoproteins, mouse c-Myc (mc-Myc) and mouse c-Jun (mc-Jun), did not differ between the livers of WT-I and mSIRT1-KI mice (Figure 7C-7F). Moreover, compared to WT-O mice, mYAP was found to be downregulated in the livers of mSIRT1-LKO mice (Figure 7G-7H). The above results were confirmed by WB experiments (Figure 7I). Interestingly, mYAP mRNA levels were also regulated by mSIRT1; mYAP mRNA was downregulated in the livers of mSIRT1-LKO mice, and upregulated in the livers of mSIRT1-KI mice, compared to WT mice (Figure 7J). hSIRT1 similarly increased human YAP (hYAP) mRNA levels in Bel-7402 and SMMC-7721 cells (Figure 7K-7L). As expected, hYAP protein levels changed in the same ways as hYAP mRNA levels in HCC cells before and after hSIRT1 overexpression and knockdown (Figure 7M-7N).

To investigate the direct link between YAP and MKK, we performed gain and loss of function studies for hYAP in Bel-7402 and SMMC-7721 cells. hYAP overexpression dramatically increased both hp-MKK3/6 and hMKK3, but not hMKK6, levels compared to the control (Figure 7O). By contrast, hYAP knockdown reduced hp-MKK3/6 and hMKK3, but not hMKK6, levels compared to the control (Figure 7P). qPCR confirmed that hYAP upregulated hMKK3 mRNA expression in HCC cells (Figure 7Q-7R). Furthermore, hYAP overexpression also increased *hMKK3* promoter activity compared to the control (Figure 7S). These results suggest that YAP may act downstream of SIRT1 to stimulate MKK3 transcription.

### Mechanism of SIRT1-induced YAP and MKK3 stimulation

**Figure 8:**
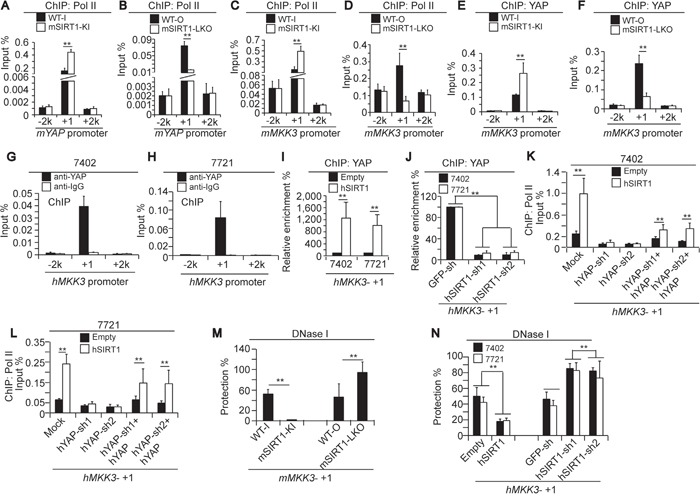
Mechanism underlying SIRT1-induced increases in MKK3 transcription via YAP **A-D.** Pol II enrichment at −2k (upstream of TSS), +1 (near the TSS), and +2k (downstream of the gene) regions of the *mYAP* and *mMKK3* promoters respectively, as measured by ChIP-qPCR in the livers of paired WT-I and mSIRT1-KI mice (A and C) or paired WT-O and mSIRT1-LKO mice (B and D). **E-F.** YAP enrichment at the −2k, +1, and +2k regions of the *mMKK3* gene in the livers of paired WT-I and mSIRT1-KI mice (E) and paired WT-O and mSIRT1-LKO mice (F), as measured by ChIP-qPCR. **G-H.** YAP occupancy at the −2k, +1, and +2k regions of the *hMKK3* gene in Bel-7402 (G) and SMMC-7721 (H) cells, as measured by ChIP-qPCR using anti-YAP antibodies. Non-specific IgG antibodies were also used as a control. **I-J.** YAP enrichment at the +1 region within the *hMKK3* promoter as measured by ChIP-qPCR in control and Bel-7402 or SMMC-7721 cells with hSIRT1 overexpression (I) or knockdown (J). **K-L.** Pol II enrichment at the +1 region of the *hMKK3* promoter in Bel-7402 (K) and SMMC-7721 cells (L) after the indicated treatments. **M.** DNase I accessibility of the +1 region of the *mMKK3* promoter in the livers of WT, mSIRT1-KI, and mSIRT1-LKO mice, as measured by CHART-PCR. **N.** DNase I accessibility of the +1 region of the *hMKK3* promoter in control and Bel-7402 or SMMC-7721 cells with either SIRT1 overexpression or knockdown, as measured by CHART-PCR. The data are shown as mean±SD from three independent experiments. **, *p*<0.01 using the Student's *t*-test.

Next, we upregulated YAP to investigate the mechanism by which SIRT1 stimulates MKK3 expression. ChIP-qPCR experiments showed that enrichment of RNA polymerase II (Pol II), a key enzyme that mediates mRNA transcription, was increased near the transcription start site (TSS, +1) regions of both the *mYAP* and *mMKK3* genes. Two additional regions 2 kilobases (kb) upstream (−2k) or downstream (+2k) of the *mYAP* and *mMKK3* genes were also tested. Pol II enrichment at the −2k and +2k regions was detectable, but low (Figure 8A-8D). These data suggest that, although Pol II may be present at sites other than the transcribed regions, it might increase elongation preferentially at the TSS. A non-specific IgG antibody was also used in parallel ChIP-qPCR experiments to determine whether Pol II binding was specific to the *mYAP* and *mMKK3* promoters. Signals indicative of Pol II binding were much higher at the −2k, +1, and +2k regions than were those generated by IgG antibodies (Supplementary Figure S1A-S1B), indicating that the ChIP signals representing Pol II binding in the gene regions were detectable above background signals. Compared to the livers of WT mice, enrichment of Pol II at the +1 regions of both the *mYAP* and *mMKK3* genes was also increased in the livers of mSIRT1-KI mice (Figure 8A and 8C) and reduced in the livers of mSIRT1-LKO mice (Figure 8B and 8D). However, no change in enrichment at either the −2k or +2k regions in either promoter was observed (Figure 8A-8D). These results suggest that SIRT1 induces Pol II-dependent transcription of both the *YAP* and *MKK3* genes.

Interestingly, YAP enrichment was also detected at the +1 region, but not the −2k or +2k regions, of the *mMKK3* promoter in mouse livers (Figure 8E-8F). Furthermore, compared to WT, mSIRT1 KI stimulated, and mSIRT1 KO suppressed, YAP enrichment at the +1 region of the *mMKK3* promoter in mouse livers (Figure 8E-8F), suggesting that SIRT1 stimulates MKK3 expression in the liver through a YAP-dependent transcription mechanism. Similarly, YAP specifically bound at the +1, but not at the −2k and +2k, regions of the *hMKK3* promoter in both Bel-7402 and SMMC-7721 cells. Moreover, ChIP experiments using non-specific IgG control antibodies showed no enrichment at the −2k, +1, and +2k regions (Figure 8G-8H). We then overexpressed and knocked down hSIRT1 to investigate whether hSIRT1 stimulates YAP binding at the *hMKK3* promoter in human HCC cells. Compared to the control, hSIRT1 overexpression induced (Figure 8I), and hSIRT1 knockdown suppressed (Figure 8J), YAP binding at the +1 region of the *hMKK3* promoter.

To determine whether SIRT1-enhanced Pol II-dependent transcription of MKK3 replies on YAP, we performed ChIP-qPCR using anti-Pol II antibodies following hYAP knockdown and overexpression. hSIRT1 overexpression induced Pol II enrichment at the +1 region of the *hMKK3* promoter compared to the empty vector-treated control; however, enrichment was not detected in hYAP-depleted Bel-7402 and SMMC-7721 cells (Figure 8K-8L). hYAP overexpression in hYAP-depleted cells restored hSIRT1-induced enrichment of Pol II at the *hMKK3* promoter compared to the empty vector-treated control (Figure 8K-8L). These results suggest that SIRT1-induced transcription of MKK3 requires YAP.

To corroborate these findings, we evaluated chromatin accessibility of the *mMKK3* locus using DNase I digestion and qPCR, a method to check chromatin status at promotes [28-29]. Indeed, the relative level of closed chromatin at the *mMKK3* promoter was significantly reduced both in human cell lines expressing ectopic hSIRT1and in mouse mSIRT1-KI cells (Figure 8M, 8N). Vice versa, in mSIRT1-LKO cells or in human cells depleted of SIRT1 by shRNAs, accessibility of the MKK3 promoter was decreased suggesting that SIRT1 modulates MKK3 expression at the chromatin level.

## DISCUSSION

Here, we showed that SIRT1 promotes cell proliferation in the livers of transgenic mouse models. SIRT1 depletion also impaired cell proliferation in human HCC cells. Both *in vivo* and *in vitro* data demonstrated that SIRT1 overexpression increases uncontrolled tumor growth in HCC. A previous study also suggested that SIRT1 expression levels are positively correlated with tumor grade, and patients with higher HCC stages tend to have higher SIRT1 expression [30]. Furthermore, patients with SIRT1-positive HCC have a lower survival rate than those with SIRT1-negative HCC [31]. In addition, depletion of SIRT1 reduced soft agar colony formation capacity in HCC cells, and xenograft growth in mice [32-33]. Collectively, data from the present and previous studies support a pro-tumorigenic role for SIRT1 in liver cancer.

We also found a positive correlation between SIRT1 and p-p38 levels, and provide evidence that p38 is a downstream effector of SIRT1 in the regulation of cell proliferation. In the absence of experimental manipulation, p38 is either not found in the nucleus or is detected in both nucleus and cytoplasm [23, 34]. After activation by phosphorylation, however, p-p38 translocates from cytoplasm to nucleus [22]. Here, we found SIRT1 did not change the subcellular localization of total-p38, which was mainly cytoplasmic. However, SIRT1 may be essential for the translocation of p-p38 to the nucleus, as SIRT1 knockout in mice and inhibition by EX527 in HCC cells reduced, while SIRT1 knock-in in mice and overexpression in HCC cells increased, nuclear accumulation of p-p38. Nuclear translocation of p-p38 may promote DNA repair [35], and we propose an additional role of nuclear p-p38 in promoting cell proliferation. Activation of p38 upregulates FLNA, which promotes cancer cell proliferation [20, 36-37]. FLNA expression was positively correlated with the expression and nuclear accumulation of p-p38 in a SIRT1–dependent manner, further supporting the importance of SIRT1 and p-p38 in stimulating HCC cell proliferation.

Pol II controls the transcription of protein-coding and microRNA (miRNA) genes in the chromatin environment, and it is part of a complex process requiring DNA-binding transcription factors, enzymes that modify histones, and other transcription co-factors that regulate loading of the Pol II machinery at gene promoters [38]. As a protein deacetylase, SIRT1 has a broad range of protein substrates, including histones and non-histone proteins [39]. SIRT1 can modulate chromatin function through deacetylation of histones, which represses transcription [38]. However, we found that SIRT1 stimulates the Pol II-dependent transcription of YAP and MKK3. In a previous study [40], we found that SIRT1-dependent deacetylation levels in histones within the *MCAM* promoter were lower in HCC cells than in other cancer cell types, suggesting that histone deacetylation may not be a major function of SIRT1 in HCC cells. SIRT1 stimulates transcription of both the *MKK3* and *YAP* genes. Interestingly, the stimulation of YAP transcription by SIRT1 was required for SIRT1-induced stimulation of MKK3 transcription, because YAP depletion greatly reduced SIRT1-induced Pol II recruitment to the *MKK3* promoter. SIRT1-mediated increases in open chromatin at the MKK3 promoter may be crucial for the function of YAP. Although there is no direct evidence that YAP can stimulate open chromatin within gene promoters, a recent study [41] showed that the oncoprotein ERG binds to YAP/TEAD-interacting regions of chromatin and increases acetylation of histone H3K9/14, a hallmark of transcription activation.

The transcription factor CREB binds to the *YAP* promoter and may stimulate YAP transcription in HCC cells [18, 42]. In the present study, we found that SIRT1 can also stimulate YAP transcription. Whether and how SIRT1 affects CREB-dependent regulation of YAP in HCC cells remains unknown. Notably, SIRT1 and CREB interact with each other in the regulation of neurogenesis [43], and SIRT1 KO mice show transcriptional defects similar to those observed in CREB KO mice [44]. Other studies have suggested that SIRT1 interacts with a wide variety of DNA-binding transcription factors and co-regulators [45]. Furthermore, deacetylation of these non-histone transcription factors by SIRT1 contributes to complex transcriptional regulation, affecting cell differentiation, metabolism, circadian regulation, stress response, and survival [38]. One study has shown that SIRT1 induces deacetylation of CREB at Lys136, suppressing of CREB activity in the regulation of lipid metabolism [46]. However, no direct evidence has shown that SIRT1 inhibits CREB activity in HCC cells, and further studies are needed to address this issue.

YAP is critical in maintaining transformative phenotypes in cancer cells [47], and hyperactivation of YAP likely contributes to mammalian carcinogenesis [48]. YAP overexpression is also closely associated with poor tumor differentiation in HCC [49]. However, Tao et al [50] concluded that overexpression of active YAP alone does not result in tumor formation in mice, while co-overexpression of YAP and β-catenin caused rapid liver tumorigenesis. Interestingly, SIRT1 increases YAP-dependent transcription in HCC cells by increasing the interaction between YAP and TEAD4, a well-characterized YAP co-transcription factor [33, 51]. In this study, we demonstrate that SIRT1 enhances YAP transcription, supporting that SIRT1 positively regulates YAP activity. Moreover, SIRT1 also deacetylates β-catenin, promoting its accumulation in the nucleus and leading to transcription of its target genes [52]. We therefore propose that SIRT1 may act as a common activator of both YAP and Wnt/β-catenin signaling during development of liver cancer.

**Figure 9:**
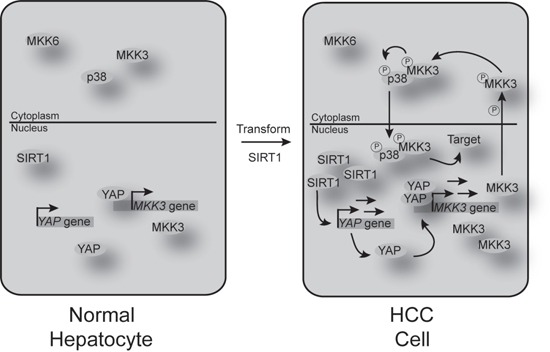
Schematic diagram of the mechanisms underlying SIRT1-induced signaling changes in HCC cells compared to normal hepatocytes

Taken together, our results demonstrate that elevated-SIRT1 expression may promote tumorigenesis in HCCs; a schematic diagram is provided in Figure 9. We have also identified a close, positive relationship between SIRT1 and p-p38 in mouse liver and human HCC cells, and shown that YAP is necessary for SIRT1-mediated activation of p-p38. Blocking both SIRT1 and YAP may prove to be an effective treatment for HCC.

## MATERIALS AND METHODS

### Mouse experiments

mSIRT1 knock-in (mSIRT1-KI, C57BL/6-*Actb^tm3.1(Sirt1)Npa^*/J) mice were purchased from the Jackson laboratory (Bar Harbor, ME, USA, #013080). Littermate C57BL/6J (WT-I) mice were used as paired controls to mSIRT1-KI mice. mSIRT1-liver knock-out (mSIRT1-LKO, Alb-Cre; mSIRT1^*loxp/loxp*^) mice were generated by crossing Albumin (Alb)-Cre mice (Shanghai Biomodel Organism Science & Technology Development Co. Ltd, Shanghai, China) with mSIRT1^*loxp/loxp*^ mice (a gift from Prof. Jun Xu, Institute of life science and technology, Tongji University, Shanghai, China). The Alb-Cre (WT-O) mice were used as paired controls to mSIRT1-LKO mice. Treatment with DEN (50 mg/kg, dissolved in saline) was initiated once mice were 12 weeks old. All animal experiments were performed with institutional approval.

### Tissue samples, cell culture, and vectors

HCC and adjacent normal liver tissues were acquired at Shanghai Ruijin Hospital with institutional approval. The HCC cell lines Bel-7402, SMMC-7721, Huh7, HepG2, SK-Hep1, and Bel-7404, and hepatocyte line HL-7702 cells, were cultured in DMEM. Cells were treated with EX527 (Selleckchem, Shanghai, China) at a final concentration of 10-50 μM and SB203580 (Beyotime, Haimen, China) at a final concentration 20 μM. The shRNAs (sh) against hSIRT1 (hSIRT1-sh1 and -sh2) and lentiviral-based hSIRT1-FLAG-expressing plasmids were purchased from Genechem Biotech LTD (Shanghai, China). The hYAP-sh1 and –sh2, lentiviral-based hYAP, and hp38 expressing plasmids were constructed in our previous studies [18, 40]. The promoter region from −1750 nt to +244 nt relative to the transcription start site (TSS) of the *hMKK3* gene was amplified via PCR from gDNA of Bel-7402 cells and cloned into pGL4.21 (Promega, Madison, WI, USA) plasmids. The primers used for this study are listed in Supplementary Table S1.

### Immunohistochemistry (IHC), immunofluorescence (IF), and Western blotting (WB)

For IHC, HCC tissue microarrays analysis (TMA) slides were purchased from Alenabio (Xi'an, China). The primary antibodies used in IHC were anti-p-p38 (Cell signaling technology (CST), Boston, MA, USA, #4511), anti-p38 (Abcam, Hongkong, China, #ab31828), anti-p-MKK3/6 (CST, #9236), anti-MKK3 (Epitomics, Burlingame, CA, USA, #1728), anti-MKK6 (Epitomics, #1640), anti-YAP (CST, #12395), anti-c-Jun (CST, #2315), anti-c-Myc (Epitomics, #1472), anti-ki67 (Abcam, #ab15580), anti-AFP (Abcam, #ab46799) and anti-SIRT1 (for mSIRT1, CST, #2028; for hSIRT1, Abcam, #ab32441). For IF, the primary antibodies used were anti-p-p38 (for mp-p38, CST, #4511; for h-p-p38, Abcam, #ab45381), anti-p38 (Abcam, #ab31828), anti-p-MKK3/6 (CST, #9236) and anti-FLAG-tag (CST, #8146). For WB, the primary antibodies used were anti-GAPDH (CST, #5174), anti-p-p38 (CST, #4511), anti-p38 (Abcam, #ab31828), anti-SIRT1 (for mSIRT1, CST, #2028; for hSIRT1, Abcam, #ab32441), anti-p-MKK3/6 (CST, #9236), anti-MKK3 (Epitomics, #1728), anti-MKK6 (Epitomics, #1640), anti-FLAG-tag (CST, #8146), anti-AFP (Abcam, #ab46799) and anti-YAP (CST, #12395). All IHC, IF and WB experiments were performed conventionally and the protocols are available elsewhere.

### Luciferase reporter, cell proliferation, chromatin immunoprecipitation (ChIP), and quantitative RT-PCR (qPCR)

Luciferase reporter, cell proliferation, and qPCR assays were performed as described previously [40]. ChIP was performed using the ChIP-IT express kit from Active Motif (Carlsbad, CA, USA). Protein-DNA complexes were incubated with 3 μg of anti-YAP (CST, #14074), anti-Pol II (Santa cruz Biotechnology, Santa Cruz, CA, USA, sc-9001), or IgG (Santa cruz Biotechnology, #sc-2345). The primers used for qPCR are listed in Supplementary Table S1.

### Co-immunoprecipitation (co-IP)

Co-IP was performed as described previously [53]. The reagents used were: protein A/G-Sepharose (Novex, Oslo, Norway), Western/IP lysis buffer (Beyotime). The anti-MKK3 (Epitomics, #1728) antibody was used for IP.

### Chromatin accessibility analysis of chromatin structure

Accessibility of DNA to digestion with DNase I (Takara, Dalian, China) was analyzed using chromatin accessibility by real-time PCR (CHART-PCR) as described previously [28]. Briefly, nuclear pellets were re-suspended in 3 ml of buffer (10 mM Tris–HCl, pH 7.4, 10mM NaCl, 3mM MgCl2, 0.3M sucrose). Aliquots of nuclei were digested with DNase I (7U) in a 100 μl final volume with 1mM CaCl2 for 5 min at room temperature. The reaction was stopped by adding 100 μl of stop buffer (50mM Tris–HCl, pH 7.5, 150mM NaCl, 50mM EDTA, 0.3% SDS) and incubated with 10 U RNase A (Takara) at 37°C for 2 h. After purification, 0.1 μg DNA from DNase I-digested or non-digested control nuclei was used in qPCR with the primer sets listed in Supplementary Table S1. Percent protection was calculated as the amount of DNA recovered from the DNase I-digested nuclei relative to the non-digested control nuclei.

### Statistical analysis

Tests used to examine the differences between groups included the Student's *t*-test and the Log Rank test. *p*<0.05 was regarded as statistically significant.

## SUPPLEMENTARY FIGURE AND TABLE


