# Supplementary material for: SIRT1 increases YAP- and MKK3-dependent p38 phosphorylation in mouse liver and human hepatocellular carcinoma
Source: Oncotarget. 2016 Jan 25;7(10):11284–98. doi: 10.18632/oncotarget.7022 (PMC4905473; doi:10.18632/oncotarget.7022)
Supplement: Supplementary file 1 [file oncotarget-07-11284-s001.pdf]

SUPPLEMENTARY FIGURE AND TABLE

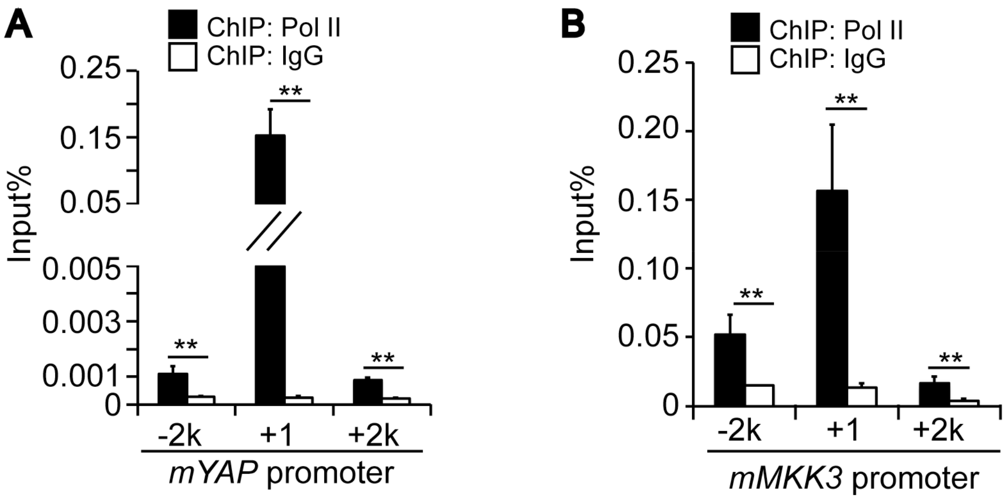

Supplementary Figure S1: Binding of Polymerase II (Pol II) at the mouse *YAP* (*mYAP*) and mouse *MKK3* (*mMKK3*) promoters. The bindings of Pol II at the +1 regions of *mYAP* **A.** and *mMKK3* promoters **B.** were measured by ChIP experiments. A 2k region upstream (-2k) of the transcription start site (TSS) or a 2k region downstream (+2k) of *mYAP* or *mMKK3* genes were also parallel analyzed as the negative controls. ChIP experiments using a non-specific IgG control were also performed to support the specific bindings of Pol II. The data are shown as the means + SD from three independent experiments. **, $p<0.01$ using the Student's t test.

Supplementary Table S1: Primers used in the study

Table S1-1 Primers for qPCR (both for regular-qPCR, CHART-PCR and ChIP-qPCR)

Name	5'-3'
ChIP-mYAP -2K-F	CACTATCACACAGCTCCTGTGTG
ChIP-mYAP -2K-R	GCTAGCTTCAGACAGGTAAGATTTG
ChIP-mYAP -+1-F	GCGCAGAGGAAGGAAGAGCGTCGAG
ChIP-mYAP -+1-R	CCCCTCTCCTCCCCTCCCCTCCGCGAGCTC
ChIP-mYAP -+2K-F	GTTTATTTGTAACTTATGTATCTG
ChIP-mYAP -+2K-R	GGGCAGCACCCGGGTGCTCCACAC
ChIP-mMKK3 -2K-F	GGGAGGCTTTTCCTGACTGCCCTC
ChIP-mMKK3 -2K-R	CTGTGGATGTAGCATTGTGGGATC
ChIP-mMKK3 -+1-F	GCCTTTTCAGGCGGGCCAATCAG
ChIP-mMKK3 -+1-R	CTAATCTAGAGGACAGCAGACTACACCG
ChIP-mMKK3 -+2K-F	GTAACAGTCAGCGCTGACCATTGGCTC
ChIP-mMKK3 -+2K-R	GCAACAGGAGACAGACACTGCTACCTC
ChIP-hMKK3 -2K-F	CTGAGTCAGTAACAAAGACATCAG
ChIP-hMKK3 -2K-R	TGTGCCAGGGATGGTCCTGCCGCAG
ChIP-hMKK3 -+1-F	AGGCAGGGACAGGCCGGCGTCTG
ChIP-hMKK3 -+1-R	GCGACGGGCGGCGCCGGAGACAGAC
ChIP-hMKK3 -+2K-F	CTGGCTGGATGCCGCAGCCCTTCAC
ChIP-hMKK3 -+2K-R	CTGTGCTTTGTAAATGTTAGTGACCTGC
mFOS-qPCR-F	CAGAAGGGGCAAAGTAGAGC
mFOS-qPCR-R	GCAACGCAGACTTCTCATCTTC
mFLNA-qPCR-F	ACTTCCCCAGCAAGCTACAG
mFLNA-qPCR-R	TGGCCCTCCAGTCTGTGTAA
mMKK3-qPCR-F	CACCTTCTATGGTGCCCTCTT
mMKK3-qPCR-R	CACAGACAGCTTGCTATGCAG
hMKK3-qPCR-F	GCATGGAGCTCATGGACACA
hMKK3-qPCR-R	AGGACATTGGAGGGCTTCAC
mGAPDH-qPCR-F	ATCATCCCTGCCTCTACTGG
mGAPDH-qPCR-R	GTCAGGTCCACCACTGACAC
hGAPDH-QPCR-F	ATCATCCCTGCCTCTACTGG
hGAPDH-QPCR-R	GTCAGGTCCACCACTGACAC

Supplementary Table S1-2: Primers used for construction of *hMKK3* promoter reporter

Name	5'-3'
hMKK3-F	ATGCGGTACCCAAGAAGCAGAGTGCACAAGGCGGA
hMKK3-R	ATGCGATATCGCGGTGGAGACTAATCTAGAGGACT
